# The Effect of Water Level in Rice Cropping System on Phosphorus Uptake Activity of *Pup1* in a *Pup1*+*Sub1* Breeding Line

**DOI:** 10.3390/plants10081523

**Published:** 2021-07-26

**Authors:** Na-Hyun Shin, O New Lee, Jae-Hyuk Han, Kihwan Song, Hee-Jong Koh, Soo-Cheul Yoo, Joong Hyoun Chin

**Affiliations:** 1 Department of Integrative Biological Sciences and Industry, Sejong University, Seoul 05006, Korea; skgus1125@gmail.com (N.-H.S.); 0724jh@gmail.com (J.-H.H.); 2 Department of Bioindustry and Bioresource Engineering, Plant Engineering Research Institute, Sejong University, Seoul 05006, Korea; onewlee@sejong.ac.kr (O.N.L.); khsong@sejong.ac.kr (K.S.); 3 Department of Plant Science, Plant Genomics and Breeding Institute, Research Institute for Agriculture and Life Sciences, Seoul National University, Seoul 08826, Korea; heejkoh@snu.ac.kr; 4 Department of Plant Life and Environmental Science, Hankyong National University, Anseong 17579, Korea; scyoo@hknu.ac.kr

**Keywords:** rice, *Pup1*, *Sub1*, P deficiency tolerance, submergence tolerance, QTL

## Abstract

Pyramiding useful QTLs into an elite variety is a promising strategy to develop tolerant varieties against multiple abiotic stresses. However, some QTLs may not be functionally compatible when they are introgressed into the same variety. Here, we tested the functional compatibility of *Pup1* and *Sub1*, major QTLs for tolerance to phosphorus (P)-deficiency and submergence conditions, respectively. Phenotypic analysis revealed that IR64-Pup1+Sub1 (IPS) plants harboring both *Pup1* and *Sub1* QTLs show significant tolerance to submerged conditions, similarly to IR64-Sub1, while IPS failed to tolerate P deficiency and mild drought conditions; only IR64-Pup1 showed P deficiency tolerance. In submerged conditions, *Sub1A* and *OsPSTOL1*, major genes for *Sub1* and *Pup1* QTLs, respectively, were expressed at the same levels as in IPS and IR64-Sub1 and in IPS and IR64-Pup1, respectively. On the other hand, in P-non-supplied condition, crown root number, root length, and *OsPSTOL1* expression level were significantly lower in IPS compared to those of IR64-Pup1. However, there was no significant difference in P content between IPS and IR64-Pup1. These results imply that *Pup1* does not compromise *Sub1* function in submerged condition, while *Sub1* suppresses *Pup1* function in P-non-supplied condition, possibly by regulating the transcript level of *Pup1*. In conclusion, *Pup1* and *Sub1* are regarded as functionally compatible under submergence condition but not under P-non-supplied condition. Further study is needed to elucidate the functional incompatibility of *Pup1* and *Sub1* QTLs in IPS under P-non-supplied condition.

## 1. Introduction

Rice (*Oryza sativa* L.) is one of the major staple food crops with high agronomic and nutritional importance, and it is widely cultivated in tropical and temperate regions of the world. Approximately, an additional 116 million tons of rice will be needed by 2035 to feed the growing population [1,2]. However, more than 40% of the rice cultivation area is subject to abiotic stresses such as phosphorus (P) deficiency, submergence, drought, and salinity. In crop improvement, pyramiding useful genes/QTLs into an elite variety is a promising strategy to develop tolerant varieties against multiple abiotic stresses. Development of a rice cultivar with tolerance to P deficiency and submergence with desirable agronomic traits is an attainable approach to address these problems [3]. 

P deficiency and submergence are considered to be serious problems limiting crop productivity throughout the world [1,4]. P deficiency in plants is often found in acidic, aluminum-toxic, manganese- or iron-toxic, drought-prone soils, high-pH soils, and upland areas [4,5,6,7]. The global demand for P fertilizer continues to increase, while global commercial phosphate reserves are estimated to deplete within the next few decades [8,9,10]. Application of P fertilizer increases cultivation cost for farmers and water pollution by fertilizer run-off. In addition, flooding is one of the most hazardous natural disasters and a major stress constraint to rice production throughout the world, especially in Southeast Asia, which results in huge economic losses. Most rice varieties will die if they are completely submerged for more than 3 days [1]. Every year, 25% of the global rice croplands are submerged by periodic flash floods, including those in Northeast Asia, which are unpredictable and can occur several times a year.

Great progress has been made during the last two decades in discovering tolerance against P deficiency and submergence in rice. Phosphorus uptake 1 (*Pup1*) is a major quantitative trait locus (QTL) located on rice (*Oryza sativa*) chromosome 12, which is associated with tolerance to phosphorus (P) deficiency in soil [11,12,13,14]. *Pup1* has been identified and cloned from an Indian landrace “Kasalath,” which was initially identified in a screening of 30 diverse rice genotypes in a P-deficient soil under rainfed conditions. Subsequently, *Pup1* introgressions showed threefold higher phosphorus uptake and grain yield than Nipponbare under P-deficient conditions [12,15,16,17]. Fine mapping identified a *Pup1*-specific protein kinase gene, *phosphorus starvation tolerance 1* (*PSTOL1*), from Kasalath BAC clones, which was not found in Nipponbare reference genome (a temperate japonica variety) and P-starvation-intolerant modern varieties. Sequencing of Kasalath BAC clones revealed a 278 kbp sequence different from the syntenic regions in Nipponbare (145 kb) and in the indica reference genome of 93–11 (742 kbp) [3]. Size differences were caused by large INDELs and an exceptionally large number of retrotransposon and transposon-related elements (TEs) in sequences. *PSTOL1* promoted crown root development and early root growth in rice under P-deficient conditions, thereby enabling plants to acquire more P and other nutrients [16]. Two dominant functional markers, K46-1 and K46-2, specific for the *Pup1* locus have been developed and used in marker-assisted selection [11]. *Pup1* is the only major QTL controlling P deficiency in plants reported up to now [10]. 

Submergence tolerance is controlled by a single major QTL on chromosome 9, along with a number of minor QTLs [18,19,20]. All these studies have used “FR13A” derived from “Dhalputtia (Indian landrace)”, which is one of the most submergence-tolerant donor varieties. The major QTL, named *Sub1*, with a LOD score of 36 and an *R*^2^ value of 69% [20], provided tolerance for up to 2 weeks after complete submergence. The fine mapping of *Sub1* employed 2950 F_2_ segregating individuals, and although the region had a low recombination rate, *Sub1* was delineated to a genomic region of approximately 0.06 cM [21]. Sequencing the *Sub1* region in an FR13A-derived line revealed the presence of three genes (*Sub1A*, *Sub1B*, and *Sub1C*), which encode putative ethylene-responsive factors (ERFs). Among the three genes, *Sub1A* was confirmed as the major determinant of submergence tolerance [22,23]. *Sub1* adversely affects grain yield by promoting ethylene genesis in the panicle at anthesis, while compromising morphological characteristics such as tiller production and stem elongation [24].

Flood areas often overlap with barren soil areas, where fertilizer deficiency and drought stress could easily occur after a flash flooding. The QTL pyramiding approach can be used by combining several important traits to develop a new breeding rice line. However, some genes/QTLs may not be functionally compatible with other genes/QTLs when they are introgressed into the same variety. Breeding a tolerant rice cultivar against P deficiency and submergence would be a challenging task because of the complexity of the component traits, screening technique, environmental factors, and genetic interactions [25]. In this study, IPS, an introgression line harboring two major abiotic-stress-tolerant QTLs (*Pup1* and *Sub1*), was developed by pyramiding, and the functional compatibility of the two QTLs in submerged and phosphorus +/− conditions was assessed.

## 2. Results

### 2.1. Genotypic Analysis of IPS

**Figure 1:**
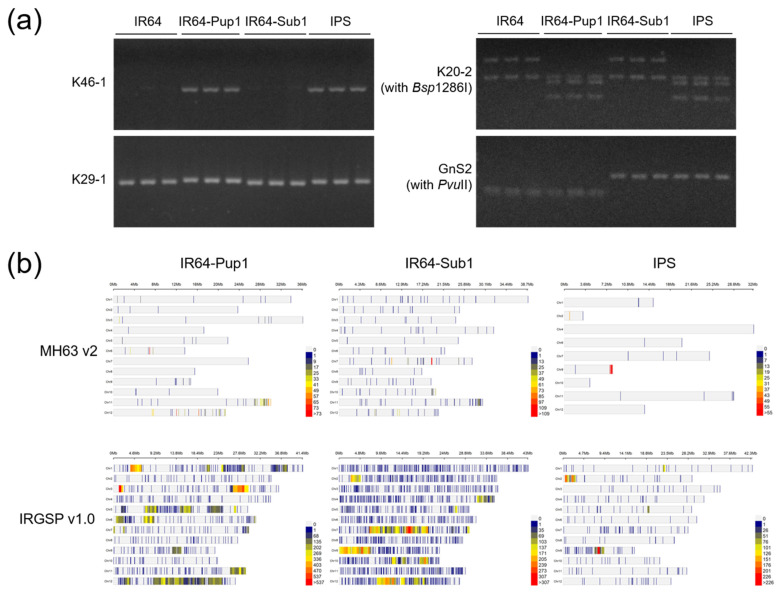
Development and selection of IR64-Pup1+Sub1 (IPS), a pyramiding line containing two major abiotic stress QTLs (*Pup1* and *Sub1*). (**a**) Foreground genotyping of IPS using *Pup1* and *Sub1* QTL/gene-specific markers. (**b**) Background genotyping using an Axiom Oryza 580 K chip set. Minghui63 v2 (indica variety) and IRGSP v1.0 (japonica variety) were used as reference genomes. The specific regions of IR64-Pup1, IR64-Sub1, and IPS in comparison with IR64 were marked on the chromosome by colored lines, respectively.

IPS, a marker-assisted gene pyramiding line, was validated using three specific markers for *Pup1* (K46-1, K29-1, and K20-2), and one for the *Sub1* (GnS2) (Figure 1a, Table S1 (Supplementary Materials)). Using PCR analysis, the tested IPS lines were confirmed to contain both *Pup1* and *Sub1*. K46-1, the *Pup1* protein kinase gene marker, identified an INDEL region of Kasalath genotypes in IR64-Pup1 and IPS. K29-1, a codominant marker that developed from the Nipponbare reference genome, showed polymorphism between lines with and without *Pup1* QTL. K20-2 marker generated three DNA fragments specific for the Kasalath allele in IR64-Pup1 and IPS, and two DNA fragments for non-Kasalath alleles in IR64 and IR64-Sub1 after digestion of PCR amplicons with *Bsp*1286I. On the other hand, GnS2, a CAPS marker to identify *Sub1* ethylene-responsive transcription factor (ERF) gene, was confirmed in IR64-Sub1 and IPS.

The IPS plants were identified by background genotyping using an Axiom Oryza 580 K chip set, which consists of SNP markers designed on the basis of Minghui63 v2 (MH63 v2; *indica* variety) and IRGSP v1.0 (*japonica* variety) (Figure 1b). The non-IR64 alleles in IR64-Pup1, IR64-Sub1, and IPS were marked on each position of the chromosome by colored lines, representing the number of polymorphism markers. The percentage of IR64 allele in IR64-Pup1 and IR64-Sub1 reached 95.8% and 97.6% based on IRGSPv1.0, and 96.3% and 96.8% based on MH63 v2. The ratio of IR64 allele in IPS was 99.7% and 99.5% based on IRGSPv1.0 and MH63 v2, respectively. The non-IR64 alleles were not detected on chromosome 3, 5, and 8 of IPS based on MH63 v2. 

### 2.2. Phenotypic Evaluation and Gene Expression of IPS in Submerged Condition 

**Figure 2:**
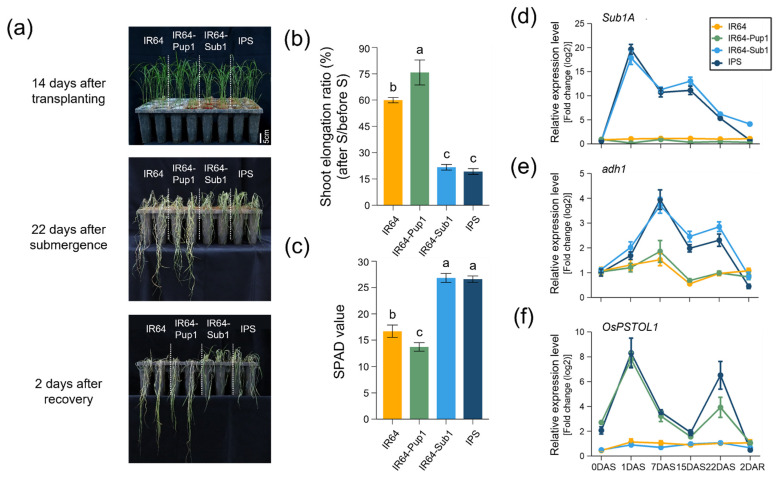
Phenotypic analysis of IR64, IR64-Pup1, IR64-Sub1, and IPS under submergence. (**a**) Phenotypic evaluation of plants after 22 days of submergence and 2 days of de-submergence. (**b**) Plant height. (**c**) The SPAD value of rice leaves. (**d**–**f**) Relative gene expression level of *Sub1A*, *adh1*, and *OsPSTOL1*, the major genes of *Sub1*, anaerobic respiration, and *Pup1*, in shoots were analyzed. Expression was calculated using relative quantification, normalized to *OsUBQ5* transcript abundance, and expressed in relation to IR64 as a calibrator sample. n = 3. The letters above the bars represent statistical significance (*p* < 0.01) as measured by Duncan’s multiple range test.

To assess the function of the *Sub1* QTL in IPS, the submergence tolerance of IR64, IR64-Pup1, IR64-Sub1, and IPS was tested by submergence quiescence ability. Plants were immersed into a 70-cm deep water tank for 22 days (22DAS), and taken out and recovered for 2 days (2DAR) (Figure 2a). IR64-Sub1 and IPS only survived with green leaves after de-submergence, whereas IR64 and IR64-Pup1 died after 22DAS. The elongation rates of plant height after submergence were significantly low in NILs harboring *Sub1* QTL. To compare the plant height before and after submergence treatment, IR64-Sub1 and IPS were increased by only 21.1% and 19.2%, respectively; however, IR64 and IR64-Pup1 were increased by 56.3% and 73.7% after submergence, respectively (Figure 2b). The *Sub1* QTL increased the chlorophyll content in the leaves after de-submergence. The soil plant analysis development (SPAD) value for *Sub1*-introgression lines (IR64-Sub1 and IPS) was higher than for others (IR64 and IR64-Pup1) under the submergence treatment. IR64 and IR64-Pup1 at 2DAR showed SPAD values of 16.69 and 13.69, whereas IR64-Sub1 and IPS showed 26.83 and 26.60, respectively (Figure 2c). 

After submergence treatment, we checked the gene expression levels of *Sub1A*, *adh1*, and *OsPSTOL1* to determine the function of *Sub1* and *Pup1* in IPS (Figure 2d–f). The expression level of *Sub1A*, a major gene of *Sub1* QTL, increased and reached a peak at 1DAS in IR64-Sub1 and IPS, and then it decreased until 22DAS (Figure 2d). At 2DAR, the expression of *Sub1A* disappeared in IPS; however, it remained at a low level in IR64-Sub1. The expression level of *adh1* (alcohol dehydrogenase 1), a gene that degrades alcohol produced by anaerobic respiration, was slightly increased in IR64 and IR64-Pup1 until 7DAS, but it was not detected after de-submergence (Figure 2e). However, the expression level of *adh1* in IPS and IR64-Sub1 was changed at the same level, which increased and reached a peak on 7DAS, and then decreased until 2DAR. *OsPSTOL1*, a major gene of *Pup1* QTL, was expressed only in IR64-Pup1 and IPS (Figure 2f), which significantly increased at 1DAS and gradually decreased until 15 DAS. Afterwards, it was temporarily increased at 22DAS and disappeared at 2DAR. *OsPSTOL1* expression was not detected in IR64 and IR64-Sub1.

In the phenotypic analysis, IPS displayed the same level of submergence tolerance as IR64-Sub1 under submergence and subsequent recovery conditions. In gene expression analysis, the expression level of *Sub1A* and *adh1* in IPS was the same as that of IR64-Sub1. Therefore, *Sub1* QTL of IPS was assumed to be performing its original function without interference from *Pup1* QTL under submergence conditions.

### 2.3. Pup1 Increased Plant Growth and P Uptake in IPS under P-Supplied Condition

**Figure 3:**
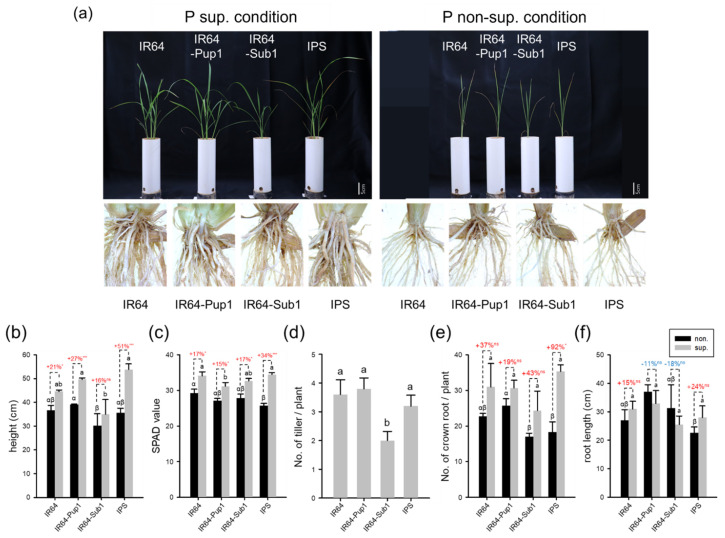
Phenotypic analysis of IPS, IR64, IR64-Pup1, and IR64-Sub1 under different phosphorus conditions 48 days after transplanting (DAT). (**a**) The phenotype of plants that were grown under P-sup. and P-non-sup. conditions, respectively. (**b**,**c**) Plant height and SPAD value. (**d**) Tiller numbers per plant under P-sup. condition. (**e**) Crown root number per plant and (**f**) root length. Colored numbers above the bars represent phenotypic change rates under P-sup. versus P-non-sup. conditions. Red/blue means an increase/decrease in each value under P-non-sup. condition. The letters above the bars indicate statistically significant differences (*p* < 0.01) as measured by Duncan’s multiple range test; values with different letters in the same-colored column are significantly different (*** significant at *p* < 0.001, * *p* < 0.05, ns: no significant difference).

To assess the function of *Pup1* QTL in soil, phenotypic analysis was conducted using a 25-cm deep pot filled with P-non-supplied soils (P-non-sup. condition; 0 g P_2_O_5_/pot) and P-supplied soils (P-sup. condition; 0.42 g P_2_O_5_/pot). The soil was maintained under mild drought condition with 80% water capacity, because *Pup1* phenotype was not observed under irrigated condition [15]. Root and shoot phenotypes were evaluated at 14 DAT (days after transplanting, Figure S1) and 48 DAT (Figure 3). The increase in plant height and SPAD value in P-sup. condition showed significant differences compared to those in P-non-sup. condition for all the NILs, except the plant height of IR64-Sub1 (Figure 3b,c). IPS showed the largest increase in plant growth in P-sup. condition compared to that in P-non-sup. condition. The plant height of IR64-Sub1 was the smallest among NILs in P-non-sup. and P-sup. conditions (Figure 3b). The plant height of IR64, IR64-Pup1, IR64-Sub1, and IPS was 44.6 cm, 49.6 cm, 38.0 cm, and 53.8 cm in P-sup. condition, which was equivalent to 121.5%, 127.1%, 115.7%, and 151.1% of the plant height in P-non-sup. condition, respectively. The SPAD value of IPS was 34.4 in P-sup. condition, corresponding to 134% of the value in P-non-sup. condition and was the highest change rate among NILs. The tiller number of IPS was more than that of IR64-Sub1, but there were no differences among IR64 and IR64-Pup1 in P-sup. condition (Figure 3d). Crown root number was the highest in IR64-Pup1 and the smallest in IR64-Sub1 in P-non-sup. condition. IPS showed the highest crown root number with a dramatic change in P-sup. condition, corresponding to 192% of that in P-non-sup. condition (Figure 3e). At 37 cm, the root of IR64-Pup1 was the longest among NILs in both P-non-sup. and P-sup. conditions, followed by IR64-Sub1 (31.3 cm), IR64 (27.0 cm), and IPS (22.6 cm) in P-non-sup. condition. IPS made big differences for plant height, SPAD value, crown root number, and root length between P-sup. and P-non-sup. conditions.

**Figure 4:**
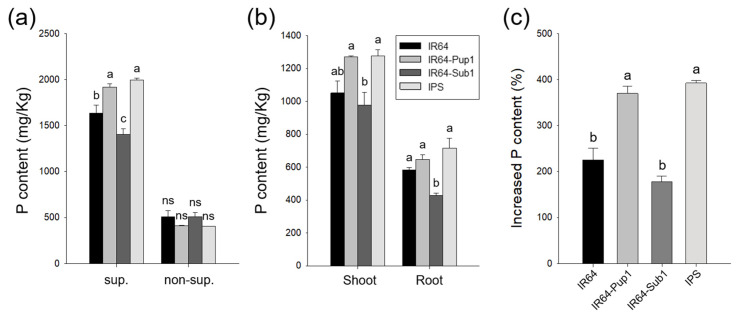
Total P content in shoot and root of IPS and NILs. (**a**) Total P content under P-sup. and P-non-sup. condition at 48 DAT. sup., P-sup. condition; non-sup., P-non-sup. condition. (**b**) Total P content of shoots and roots under P-sup. condition. (**c**) Increased ratio of total P content under P-non-sup. condition versus P-sup. condition. The letters above the bars indicate statistically significant differences (*p* < 0.01) as measured by Duncan’s multiple range test. ns: no significant difference.

The total P content in the whole plant of *Pup1*-introgression lines (IR64-Pup1 and IPS) was significantly higher than that of *Pup1*-non-introgression lines (IR64 and IR64-Sub1) in P-sup. condition, while there were no differences among NILs in P-non-sup. condition (Figure 4a). In addition, the total P content in the shoots and roots of *Pup1*-introgression lines (IR64-Pup1 and IPS) was higher than for IR64-Sub1 (Figure 4b). The increased P content ratio of P-sup. condition to P-non-sup. condition was 392.4% and 369.8% for IPS and IR64-Pup1, respectively, which was significantly higher than that of IR64 (224.9%) and IR64-Sub1 (177.2%) (Figure 4b,c). From these results, *Pup1* was assumed to be functioning compatibly with *Sub1* to increase P uptake in the soil system under sufficient phosphorus conditions.

### 2.4. Interaction of Pup1 with Sub1 Repressed OsPSTOL1 Expression in IPS under Mild Drought Condition

**Figure 5:**
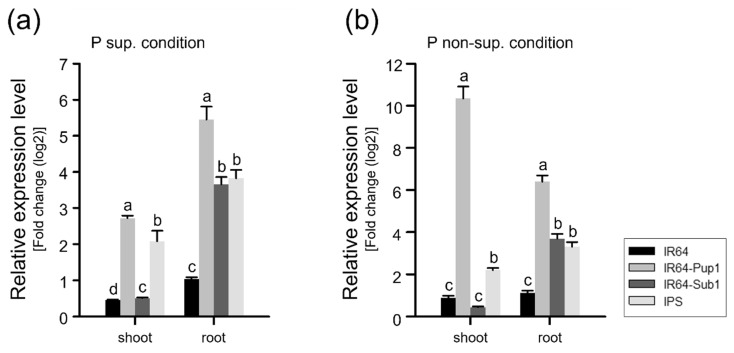
Relative expression levels of *OsPSTOL1*, a major gene of *Pup1* QTL, in shoots and roots under P-sup. condition (**a**) and P-non-sup. condition (**b**) 14 days after transplanting (DAT). The expression level was calculated using relative quantification, normalized to *OsUBQ5* transcript abundance, and expressed in relation to IR64 as a calibrator sample. n = 3. The letters above the bars indicate statistically significant differences (*p* < 0.01) as measured by Duncan’s multiple range test. ns: no significant difference.

The gene expression level of *OsPSTOL1* was analyzed for all NILs grown under P-sup. and P-non-sup. conditions (Figure 5a,b). Plants were grown under mild drought condition to assess *Pup1* function properly. We used 14-day-old plants for RNA extraction since the RNA transcription level increased prior to the morphological changes. The *OsPSTOL1* expression level in *Pup1*-introgression lines (IR64-Pup1 and IPS) was higher than that in *Pup1*-non-introgression lines (IR64 and IR64-Sub1) in both P-sup. and P-non-sup. conditions. The *OsPSTOL1* expression was detected in the roots of IR64-Sub1 under both P conditions; however, the amplicon turned out to be false positive by agarose gel electrophoresis (Figure S2). IR64-Pup1 displayed the highest expression level in the shoots and roots in both P-sup. and P-non-sup. conditions (Figure 5a,b). The expression levels in the shoots and roots of IR64-Pup1 were 2.67 and 5.45 in P-sup. condition, but 10.35 and 6.50 in P-non-sup condition, respectively. *OsPSTOL1* expression in the shoots of IR64-Pup1 was significantly higher than in other NILs in P-non-sup. condition. The *OsPSTOL1* expression level of IPS was significantly lower than that of IR64-Pup1 in both shoots and roots, regardless of P conditions. It was assumed that the *Sub1* QTL or introgressed segment interfered with the function of *Pup1* QTL of IPS under mild drought condition.

## 3. Discussion

Rice is the most important staple food for more than half of the world’s population. As the world population is predicted to reach 9.6 billion by 2050, there is a strong imperative to increase rice production to meet the growing global food demand [26]. However, there is currently more abiotic and biotic stress caused by climate change and increased competition for scarce resources such as land and water [27]. Here, we developed IR64-Pup1+Sub1 (IPS) by introgression of two abiotic-stress-resistant QTLs of *Pup1* and *Sub1* into elite-variety IR64, which imposes constraints on plant growth and production of rice (Figure 1). 

Submergence is a recurring problem in the rice-producing rainfed lowlands of South and Southeast Asia. *Sub1* is a major QTL conferring tolerance on flash floods or 1–2 week submergence, and it improves rice varieties grown in rainfed lowland and irrigated areas. In a previous study, *Sub1* introgressions into the two popular rice cultivars (Swarna and Savitri) were reported to increase susceptibility to stagnant flooding [24]. Phenotypic analysis revealed that IPS plants show significant tolerance to submerged conditions, similarly to IR64-Sub1, while *Sub1* non-introgression lines (IR64 and IR64-Pup1) displayed rapid plant growth, which led to drying up under submergence condition (Figure 2). The expression level of *Sub1A* and submergence resistance were closely related in NILs. The elongation rates of plant height after submergence for *Sub1*-introgression lines (IPS and IR64-Sub1) were significantly lower than those for non-introgression lines. This seemed to be related with a quiescence strategy of *Sub1A* plants, which allows them to reduce carbohydrate use to the minimum level required to keep the plant alive, and stops growth until the flood subsides [1,28,29].

P deficiency is a major problem in upland or drought-prone rainfed lowlands [17,30]. P deficiency can affect cell wall extensibility and turgor pressure, because it leads to low ATP concentrations [31]. P deficiency commonly stimulates root elongation and root hair length and density to improve P uptake through expansion of root surface area [30,32,33]. *Pup1* QTL was discovered to be related to a high P uptake, an increase in tillering ability, and an improvement in crown root number and root elongation under P-deficient conditions [4,6,7,12,17]. In this study, IR64-Pup1 showed P deficiency tolerance with an increase in plant height, crown root number, and root length under P-non-sup. condition (Figure 3). *Pup1* introgressed and non-introgressed lines showed a reduction in overall growth under P-non-sup. condition, which seemed to be related with the lower rate of tissue expansion due to a decrease of cell division with higher cell production rates [34]. 

Flooding the soil tends to increase the availability of phosphorus (P) to plants, because of faster diffusion to roots and increase in phosphorus solubility as a result of reductive dissolution of iron oxides [35]. In the present study, *OsPSTOL1*, a major gene of *Pup1* QTL, was expressed in IR64-Pup1 and IPS, which significantly increased in the early stage of submergence and gradually decreased during submergence (Figure 2). This suggests that *OsPSTOL1* expression facilitates the uptake of phosphorus in the early stage of submergence. However, if oxygen deficiency occurs after continuous submergence, P uptake could be inhibited by reduced *OsPSTOL1* expression. 

**Figure 6:**
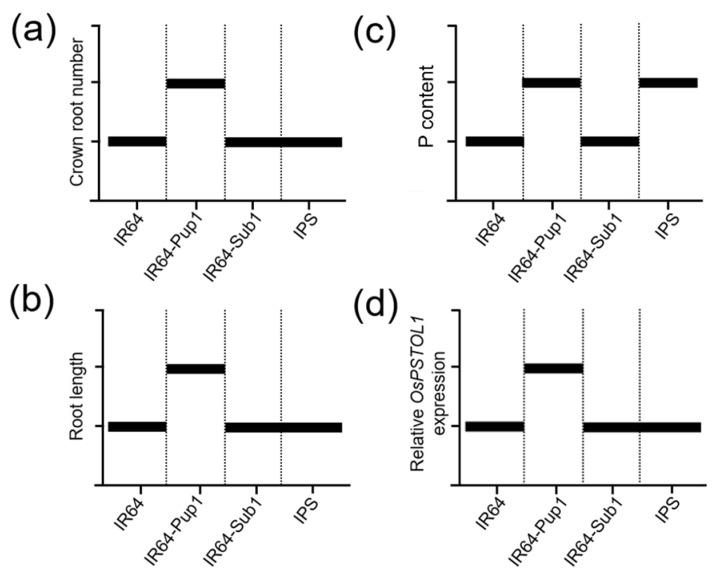
Schematic illustration of functional compatibility between *Sub1* and *Pup1* in IPS. The graphs were plotted with relative changes of phenotypic traits and gene expression level to IR64 (control). (**a**) Crown root number per plant; (**b**) root length; (**c**) P content; and (**d**) relative *OsPSTOL1* expression level in IPS, IR64, IR64-Pup1, and IR64-Sub1 in P-non-sup. condition at 48 DAT.

IPS was sensitive to phosphoric acid concentrations compared to IR64-Pup1 and other NILs, which showed significant changes in plant growth in P-sup. condition. Plant height, crown root number, and root length of IPS in P-sup. condition were 151%, 192%, and 124% compared to those in P-non-sup. condition, respectively. In P-sup. conditions, there were no significant differences in phenotype and P content between IR64-Pup1 and IPS; however, the expression level of *OsPSTOL1* was reduced in IPS compared to that in IR64-Pup1. Therefore, *Pup1* was estimated to perform the function of P uptake, but it did not interrupt plant growth under P-sup. condition. On the other hand, in P-non-sup. condition, root phenotypes (crown root number and root length) were significantly reduced in IPS compared to those in IR64-Pup1 (Figure 3 and Figure 6). IPS did not show the root phenotype indicating resistance to phosphorus deficiency in P-non-sup. condition, such as increase in crown root number and root length in IR64-Pup1. However, there was no significant difference in P content between IR64-Pup1 and IPS, which is presumably related with the limited availability of phosphorus to plants in the soil. The expression level of *OsPSTOL1* was significantly different and lower in IPS compared to expression in IR64-Pup1 in P-non-sup. conditions. Thus, it was assumed that the *Pup1* function related to phosphorus utilization may have been blocked or interfered with by *Sub1A* or *Sub1A*-linked genes in P-non-sup. condition. From these results, *Pup1* does not compromise *Sub1* function in submerged condition, while *Sub1* suppresses *Pup1* function in P-non-sup. condition, possibly by regulating the transcript level of *Pup1*, although P content was at a level similar to that of IR64-Pup1. To clarify these things, transcriptome analysis is currently being conducted.

The identification of important, QTL-controlled agricultural traits has been difficult because of their complex inheritance; however, completion of the rice genomic sequence has facilitated the cloning of QTLs and their pyramiding for breeding. Because QTLs are derived from natural variation, the use of a wider range of variations such as that found in wild species is important. Polygenic traits governed by more than one gene within the identified QTLs do not follow the simple rule of single gene introgression [36]. The positive/negative interactions of alleles within QTLs and with the genetic background, pleiotropic effect of genes, and linkage drag play an important role in determining the effect of introgressed loci [37,38,39,40,41,42,43,44]. The reported linkage drag of the QTLs has been successfully broken, and individual QTLs have been introgressed into improved genetic backgrounds using closely linked markers [41]. To identify an appropriate number of plants with positive interactions and high phenotypic expression, MAB requires genotyping and phenotyping of large numbers of plants/progenies in each generation. The population size to be genotyped and phenotyped for complex traits such as abiotic stresses increases significantly as two or more QTLs are considered for introgression. IPS with a limited introgression size of *Sub1* allele at the target region needs to be tested to prevent any negative linkage drag, which information is important to pyramid *Sub1* with other traits. 

A kind of “match-making” strategy to acquire multiple stress tolerant traits in plant breeding systems could be established by precise pyramiding using gene-specific molecular markers. In our study, the two QTLs, found to be highly effective under the corresponding stressed conditions, did not show the additive effect when they were combined into one plant, say “well matched.” Accumulating more data under various conditions is a prerequisite when we combine multiple traits to overcome the advent of climate and resource crises.

## 4. Conclusions

We developed IR64-Pup1+Sub1 (IPS) by pyramiding two QTLs controlling tolerance against P deficiency (*Pup1*) and submergence (*Sub1*) in IR64. *Pup1* does not compromise *Sub1* function in submerged condition, while *Sub1* suppresses *Pup1* function in P-non-sup. condition. In conclusion, *Pup1* and *Sub1* are regarded as functionally compatible under submergence condition but not under P-non-sup. condition. Further study is needed to elucidate the genetic network on the functional incompatibility of *Pup1* and *Sub1* QTLs in IPS under P-deficient condition.

## 5. Materials and Methods

### 5.1. Plant Materials

IR64, IR64-Pup1, and IR64-Sub1-AG1 (ISA) were introduced to Sejong University (SJU) by the Seconded Special Material Transfer Agreement (seconded SMTA) with Hankyong National University (HKNU). In the process of hybridization of IR64-Pup1 and ISA, the plants containing only *Sub1* and *Pup1* were selected and used in this study as IR64-Pup1+Sub1 (IPS). IPS, IR64, IR64-Pup1, and IR64-Sub1 were tested under different phosphorus-concentrated soils and under submerged conditions. IR64-Pup1 and IR64-Sub1 were used as tolerant lines under phosphorus and submergence stress conditions, respectively. 

### 5.2. Genomic DNA Extraction and Genomic Analysis 

Genomic DNA of IR64, IR64-Pup1, IR64-Sub1, and IPS was extracted using the cetyl-trimethyl-ammonium bromide (CTAB) method [45]. Two-week-old leaves of the testing plants were used for genomic DNA extraction. To check the introgression of *Pup1* and *Sub1*, foreground selection was performed by PCR with QTL/gene-specific markers, which were referred to in [16,23]. PCR reaction volume was 20 µL (IN5001-0500, Inclone, Yongin, Korea) and included 100 ng of template DNA and 10 pmole of forward and reverse primers (Bioneer, Dajoen, Korea). The PCR cycling conditions were followed as follows: initial denaturation for 2 min at 95 °C, followed by 35 cycles of denaturation at 95 °C for 20 s, annealing at 55–58 °C for 30 s, extension at 72 °C for 40–60 s, with a final extension at 72 °C for 5 min. PCR amplification was conducted in a PCR thermocycler (SimpliAmp, Thermo Scientific, Waltham, MA, USA). The amplified products were electrophoresed (BioFACT, Daejeon, Korea) in 2–4% agarose gel and visualized using a gel documentation system (GDS 200, Korea Lab Tech, Seongnam, Korea). The primers and sequences used in the study are listed in Table S1.

Genome background analysis was conducted using Axiom_Oryza_580K_chipset [46]. Axiom_Oryza_580K_chipset consists of 500,725 SNP markers, which were designed based on IRGSP v1.0, and 22,820 SNP markers, which were designed based on MH63 v2. With chipset data, each NIL (IR64-Pup1, IR64-Sub1, and IPS)-specific SNP was selected and marked at the position of each chromosome using linMap in R package. 

### 5.3. Submergence Stress Sensitivity Screening System

The submergence screening was conducted in a greenhouse under paddy field conditions and a plate with 32 pots (w × l × h: 56 × 28 × 17 cm) was used. Each pot has four holes in the vertical corner, so the thin felt (w × l: 23 × 23 cm) was placed in the pot to prevent loss of soils. The pots were filled with the same volume of soils and placed in the plastic box (w × l × h: 59 × 38 × 15 cm) filled with 10 cm deep water for one day to soak the soils. Seeds of IPS, IR64, IR64-Pup1, and IR64-Sub1 were sterilized for 24 h and incubated in the dark at 28 °C to germinate. Two pre-germinated seeds were sowed in each pot after two days and were covered with 1 cm deep soils. The plants were grown for 14 days under normal conditions. Plant height and SPAD value were measured with 35 plants at 14 DAT, which was regarded as 0 DAS. 

### 5.4. Phenotyping under Two Different Phosphorus Concentration Conditions in Soils

Seeds of IPS, IR64, IR64-Pup1, and IR64-Sub1 were sterilized in 50 mL of disinfectants at 28 °C for 24 h. The washed seeds were transferred to a clean petri dish containing filter paper, and 10 mL of distilled water was added and sealed with parafilm to prevent evaporation. The seeds were incubated in darkness at 28 °C for two days. To build a stable soil environment, the soils air-dried in the sun were homogenized with a sieve of 2 mm holes. Cylinder-shaped pots (r × h: 3.75 × 25 cm) were used to assess the *Pup1* function. The pots contain two holes with 1 cm diameter for drainage, and the holes are spaced 1 cm apart from the bottom for drainage. Thin aluminum plates (w × l × t: 23 × 5 × 0.05 cm) having 3.0 mm holes were placed inside the pots to prevent soil loss. The soils were placed in pots with 18 cm depth and flattened, and 0.52 g of N and 0.35 g of K_2_O were treated on the surface of the soils in each pot as a basal fertilization. P-non-supplied soils (P-non-sup. condition; 0.0 g P_2_O_5_/pot) and P-supplied soils (P-sup. condition; 0.42 g P_2_O_5_/pot) were designed. Mineral contents of the soils were analyzed (Table S2). Fertilizers were mixed with 5 cm deep soils. The pots were placed in the box filled with water to let the soil soak the water completely for three days. To create the mild drought condition, each pot was weighed before and after soaking as a zero and full point of field capacity. The water content of each pot was maintained at a level of 80% of weight gap. The weights of the pots were measured every three days, and the weight was maintained by additional watering through the bottom holes. The two pre-germinated seeds were sowed on the top of soils and covered with 1 cm deep soils. After one week, one plant in the pots had thinned. Phenotyping was performed twice, 2 weeks after transplanting (2 WAT) and 7 weeks after transplanting (7 WAT). The height, SPAD value, and tiller number per plant of 11 and 5 plants were measured at 2 and 7 WATs. Three plants whose plant height and tiller number per plant are the closest to the average value were selected to analyze the root phenotype. The roots were washed carefully, and the root length was measured. The crown root number was counted with the picture that was taken of a part where the root emerged. After phenotyping, three plants were divided into shoot and root parts and bulked to analyze the total P contents. Bulk samples were dried in a dry oven at 65 °C for 2 weeks. The total P content was analyzed at NICEM (National Instrumentation Center for Environmental Management; Seoul National University, Seoul, Korea) using an inductively coupled plasma atomic emission spectrometer (ICP; ICP-730ES, Varian, Australia).

### 5.5. RNA Extraction and Analysis of Transcriptional Levels

Seedlings of IPS, IR64, IR64-Pup1, and IR64-Sub1 were used to analyze the expression levels of *Sub1A*, *adh1*, and *OsPSTOL1* under submergence stress. The plants were divided into shoot and root parts and sampled, separately, and three of each line were harvested at sampling points. Fourteen-day-old plants were harvested at 0 DAS (days after submergence). During submergence, sampling was performed at 1, 4, 7, 15 and 22 DAS. After being de-submerged, the plants were harvested at 2 DAR (days after recovery) as a final sample. In phosphorus treatment, 14-day-old seedlings were used for analyzing the transcriptional level of *OsPSTOL1* in P-sup. and P-non-sup. conditions. At each sampling point, three plants were harvested. The plants were rinsed in tap water, and the remaining water droplets on the surface were removed with a paper towel. The washed plants were divided into shoot and root parts, respectively, and frozen in liquid nitrogen. RNA extraction was conducted using TRIzol following the manufacturer’s protocol (Thermo Fisher Scientific, MA, USA), and 100 µL of first-strand cDNA was synthesized as 1000 ng/µL using Easy cDNA Synthesis Kit (Nanohelix, Daejeon, Korea) with five times dilution. A real-time PCR mixture was made using SensiFAST SYBR No-Rox Kit (Bioline, Meridian, London, UK) and real-time PCR conducted with 20 µL of the mixture with 10 ng cDNA and 10 pmole of FW and RV primers (Bioneer, Daejeon, Korea). Real-time PCR was conducted using CFX Connect (Biorad, Hercules, CA, USA) under the following conditions: the initial denaturation at 95 °C for 15 min, followed by 40 cycles of denaturation at 95 °C for 10 s, annealing at 61 °C for 15 s, and elongation at 72 °C for 20 s. The melt curve was drawn with a 0.5 °C increment for 5 s at each temperature from 65 °C to 95 °C. The real-time PCR results were normalized to *OsUBQ5* and analyzed following the 2^−^^ΔΔ^^Ct^ method [14,47]. The primers and sequences used in this study are listed in Table S1. Duncan’s test was conducted using R version 3.6.3 (Rstudio, Boston, MA, USA). 

## Data Availability

Not applicable.

## Supplementary Materials

The following are available online at https://www.mdpi.com/article/10.3390/plants10081523/s1: Table S1: List of primers used in this study; Figure S1: Phenotypic analysis of IR64, IR64-Pup1, IR64-Sub1 and IPS under different phosphorus conditions at 14 days after transplanting (DAT); Figure S2: The confirmation of quantitative PCR amplicons of *OsPSTOL1*; Table S2: Analysis of mineral properties in soil.
