# Supplementary material for: The Effect of Water Level in Rice Cropping System on Phosphorus Uptake Activity of Pup1 in a Pup1+Sub1 Breeding Line
Source: Plants (Basel). 2021 Jul 26;10(8):1523. doi: 10.3390/plants10081523 (PMC8402110; doi:10.3390/plants10081523)
Supplement: Supplementary file 1 [file plants-10-01523-s001.zip › Figure S1.pdf]

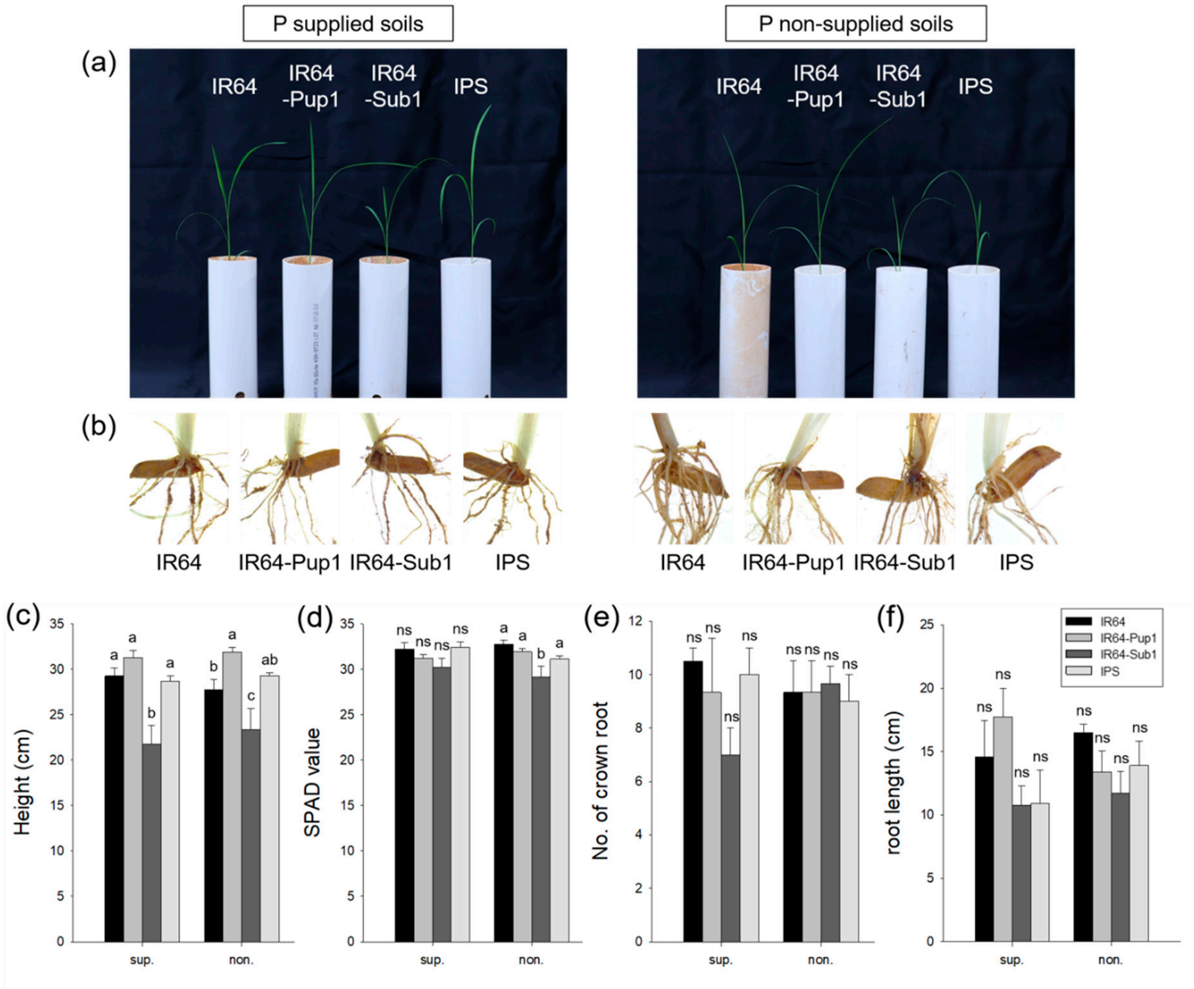

Figure S1. Phenotypic analysis of IR64, IR64-Pup1, IR64-Sub1, and IPS under different phosphorous conditions at 14 days after transplanting (DAT). (**a**,**b**) Phenotype of plants that were grown under P supplied and P non-supplied conditions, respectively. (**c**,**d**) Plant height and SPAD value. (**e**) Tiller numbers per plant under P-supplied condition. (**f**,**g**) Crown root number per plant, root length. The letters above bars represent statistical significance ($p < 0.01$) as measured by Duncan's multiple range test.
