# Supplementary material for: The Effect of Water Level in Rice Cropping System on Phosphorus Uptake Activity of Pup1 in a Pup1+Sub1 Breeding Line
Source: Plants (Basel). 2021 Jul 26;10(8):1523. doi: 10.3390/plants10081523 (PMC8402110; doi:10.3390/plants10081523)
Supplement: Supplementary file 1 [file plants-10-01523-s001.zip › Figure S2_20210722.pdf]

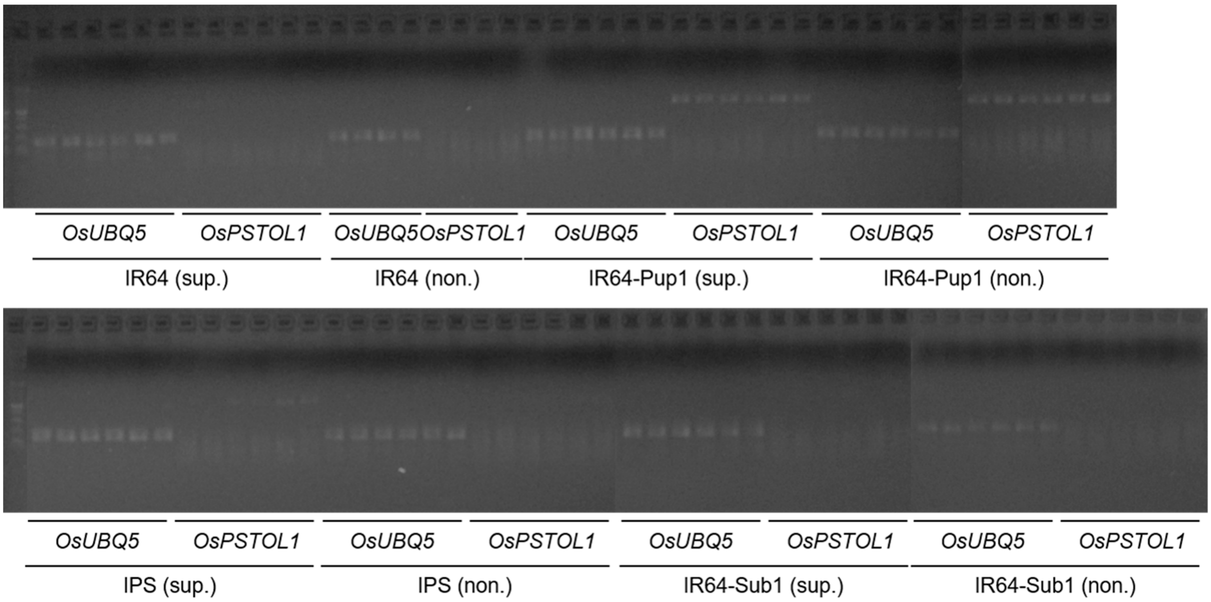

Figure S2. The confirmation of quantitative PCR amplicons of *OsPSTOL1* in IR64, IR64-Pup1, IPS, and IR64-Sub1 in P supplied and non-supplied condition. *OsUBQ5* was used as an internal control of rice to normalize gene expression in real-time RT-PCR experiments.
